# Immune-Related Circulating miR-125b-5p and miR-99a-5p Reveal a High Recurrence Risk Group of Pancreatic Cancer Patients after Tumor Resection

**DOI:** 10.3390/app9224784

**Published:** 2019-11-08

**Authors:** Eveline E. Vietsch, Ivana Peran, Mustafa Suker, Thierry P. P. van den Bosch, Fleur van der Sijde, Johan M. Kros, Casper H. J. van Eijck, Anton Wellstein

**Affiliations:** 1 Department of Oncology, Lombardi Comprehensive Cancer Center, Georgetown University, Washington, DC 20007, USA; 2 Department of Surgery, Erasmus MC, University Medical Center Rotterdam, 3015GD Rotterdam, The Netherlands; 3 Department of Pathology, Erasmus MC, University Medical Center Rotterdam, 3015GD Rotterdam, The Netherlands

**Keywords:** pancreatic cancer, resection, circulating microRNAs, biomarkers, progression free survival, immune cells

## Abstract

Clinical follow-up aided by changes in the expression of circulating microRNAs (miRs) may improve prognostication of pancreatic ductal adenocarcinoma (PDAC) patients. Changes in 179 circulating miRs due to cancer progression in the transgenic *Kras*^G12D/+^; *Trp53*^R172H/+^; *P48-Cre* (KPC) animal model of PDAC were analyzed for serum miRs that are altered in metastatic disease. In addition, expression levels of 250 miRs were profiled before and after pancreaticoduodenectomy in the serum of two patients with resectable PDAC with different progression free survival (PFS) and analyzed for changes indicative of PDAC recurrence after resection. Three miRs that were upregulated ≥3-fold in progressive PDAC in both mice and patients were selected for validation in 26 additional PDAC patients before and after resection. We found that high serum miR-125b-5p and miR-99a-5p levels after resection are significantly associated with shorter PFS (HR 1.34 and HR 1.73 respectively). In situ hybridization for miR detection in the paired resected human PDAC tissues showed that miR-125b-5p and miR-99a-5p are highly expressed in inflammatory cells in the tumor stroma, located in clusters of CD79A expressing cells of the B-lymphocyte lineage. In conclusion, we found that circulating miR-125b-5p and miR-99a-5p are potential immune-cell related prognostic biomarkers in PDAC patients after surgery.

## 1. Introduction

Pancreatic ductal adenocarcinoma (PDAC) is expected to become the second most frequent cause of cancer death by 2030 [1]. Although PDAC can be resected in early stage disease, the five-year survival rate of patients that undergo surgery is less than 17% [2–5]. Adjuvant chemotherapy after surgery is indicated, however approximately half of the patients are not able to receive adjuvant chemotherapy due to health deterioration postoperatively [6]. Also, rapid development of metastases shortly after removal of the primary tumor occurs in a subset of PDAC patients. Clinical surveillance after resection is performed through physical examination and computed tomography (CT) imaging upon indication, whereas molecular markers of PDAC progression during follow-up remain underexplored. Since collection of tissue biopsies from the pancreas is difficult and expensive, minimally invasive biomarkers attainable as ‘liquid biopsies’ from blood draws are sorely needed to aid in prognostic stratification of patients and possible adjustment of the treatment regimen. Here, we describe circulating cell free non-coding nucleic acids as potential prognostic biomarkers.

Mature microRNAs (miRs) are highly conserved short strands of non-coding RNAs that regulate gene expression. To date, more than 2600 human mature miRs have been identified and annotated [7] and more than half of human protein-coding genes are likely regulated by at least one miR [8]. MiRs are dysregulated in cancer and play crucial roles in immune function, cell proliferation, apoptosis, metastasis, angiogenesis, and tumor–stroma interactions [9–11]. It is noteworthy that miRs released from cells can induce miR-mediated gene expression alterations in neighboring as well as in distant cells when entering the circulation [12,13]. In the circulation, miRs are relatively stable and easy to measure, which has inspired a vast amount of biomarker research. The majority of research on circulating miR signatures in oncology is focused on diagnostics [14–18], however miRs can provide crucial insights into cancer progression and the effects of therapeutic interventions [19–22]. In the present study, we profiled serum miRs in a transgenic mouse model of PDAC progression and in PDAC patients with early and late disease progression, using blood samples before and after tumor resection, to identify novel circulating biomarkers of PDAC progression.

## 2. Materials and Methods

### 2.1. Blood Collection and Serum miR Analysis in KPC Mice

The experiment with genetically engineered *LSL-Kras*^G12D/+^; *LSL-Trp53*^R172H/+^; *P48-Cre* (KPC) mice [23] in this study was approved by the Georgetown University Institutional Animal Care and Use Committee (IACUC). Twelve KPC mice were euthanized at the age of 5 months before ~1 mL blood was collected via cardiac puncture in Serum Z-Gel tubes with clotting activator (Sarstedt). The serum tubes were inverted 5 times and centrifuged at 10,000 × *g* for 15 min. The serum was stored in aliquots at −80 °C until further analyses. In addition, pancreas, liver and lungs were collected and processed by formalin fixation and paraffin embedding (FFPE). Tissues were stained with hematoxylin and eosin (H&E) and the slides were examined by a clinical pathologist to evaluate pancreatic neoplasia and to determine the tumor stages. Two sections of the entire pancreas were randomly selected. Multiple tissue sections from the liver and lungs were evaluated to detect micrometastatic lesions. The animals were then divided into two groups based on disease progression: one group with PanIN-3 lesions as the worst disease stage (*n* = 3), and a second group of mice with PDAC as well as lymph node, liver, and lung metastases (*n* = 3). The remaining animals had local PDAC only (*n* = 6), and were not included in our miR study.

Equal volumes of serum samples from the mice belonging to the same group were pooled together, followed by miR isolation of the two samples, using the miRCURY RNA Isolation Kit for Biofluids (Exiqon). The murine miRs were reverse transcribed to complementary DNA (cDNA) using the miRCURY LNA™ Universal RT microRNA PCR, Polyadenylation and cDNA synthesis kit II (Exiqon). Expression of 179 miRs was analyzed with the qPCR-based Serum/Plasma Focus microRNA PCR Panel (Exiqon) using the ExiLENT SYBR^®^ Green master mix (Exiqon). MiRs with raw threshold cycle (Ct) values higher than 30 cycles were excluded. The median miR expression value in each pooled serum sample was used to normalize for miR expression. The fold differential expression for each miR was calculated (2ˆ-ΔΔCt) and plotted with Prism Graphpad 5.01, using ≥3-fold differences as the cutoff.

### 2.2. Patient Blood Collection

The patients provided written informed consent for participation and the protocols associated with this research were approved by the Erasmus Medical Center Medical Ethical Committee. Peripheral venous blood samples were obtained at the Erasmus Medical Center in the Netherlands from treatment-naive patients with resectable PDAC, one day before pancreaticoduodenectomy and 4 weeks (range 2–6 weeks) after resection. Patients who had prior gastro-intestinal malignancies were excluded. For each serum sample, a total of 8.5 mL of venous blood was collected in SST II Advance serum tubes (BD) with clot activator of silica particles to induce coagulation. After inverting the tubes six times, the samples were spun within 4 h after blood draw at 1258 × *g* for 10 min at 4 °C in a swing-bucket centrifuge (Eppendorf 5810R). The serum was divided into 1 mL aliquots and stored at −80 °C until further analyses.

### 2.3. Patient Serum miR Quantitation

Cell-free circulating miRs from the patients were isolated from 200 μL serum using the miRNeasy serum/plasma miRNA Isolation Kit (Qiagen) and eluted in 15 μL nuclease free water. Two proprietary pre-mixed spike-in ~20 nt control RNAs from MiRXES, Singapore, with sequences distinct from annotated mature human miRNAs (miRbase version21) were added into the lysis buffer prior to sample miR isolation, in order to evaluate RNA isolation efficiency. MiRs were reverse transcribed using IDEAL miR-specific oligos in a multiplex reaction per manufacturer’s instruction (MiRXES). In brief, up to 2 μL sample RNA were mixed with 1 μL RT Spike-in RNA, RT Buffer, nuclease free water, reverse transcriptase enzyme and a maximum of 10 different miR-specific RT oligos into 20 μL reactions and incubated at 42 °C for 30 min followed by heat inactivation at 95 °C for 5 min in a SimpliAmp thermal cycler (Applied Biosystems). cDNA was stored at −20 °C (up to 4 weeks) and thawed only once. Before miR quantitation, the cDNA was diluted 1:10 in nuclease free water.

For the Reverse Transcription quantitative PCR (RT-qPCR), 5 μL of sample cDNAs were mixed with the individual miRNA qPCR Assays (MiRXES), nuclease free water and the IDEAL miRNA qPCR Master Mix containing the passive reference dye ROX, into reactions of 20 μL volume. The IDEAL assay from MiRXES utilizes miR-specific reverse transcription, as well as a combination of a miR-specific forward and reverse primer to quantitate miR expression, described by Wan G. et al. [24], allowing for miR-specific and sensitive PCR. The following protocol in the 7500 Fast Real-Time PCR System (Applied Biosystems) was used: 10 min at 95 °C, 5 min at 40 °C, followed by 40 cycles of: 10 s at 95 °C and 30 s at 60 °C with FAM fluorescence reading at the end of this step. The Ct values were determined using the 7500 Software (Applied Biosystems). Technical variations introduced during RNA isolation and the process of RT-qPCR were normalized using the measurements of the spike-in control RNAs.

In the first screen, 250 miRs were measured in the serum before and after surgery of *n* = 2 patients: one with early progressive disease after 7 months, and one patient with late disease progression after 18 months. The Ct value cutoff was 33 cycles to ensure reliability of the miR measurements. MiR levels were normalized using interplate calibrators, two spike-in control RNAs and the average expression value of two stably expressed reference miRs (miR-29c-5p and miR-421). The reference miRs were selected based on their stability factor, previously described by others [25,26]. MiRs that were altered ≥3-fold after tumor resection, in opposite direction between the patients with short versus long progression free survival (PFS) were compared to the differentially expressed metastases-related miRs of the KPC mice. The miRs that were upregulated in progressive PDAC in mice as well as in patients, plus the two reference miRs were measured in the serum of *n* = 26 additional patients with resectable PDAC before and after pancreaticoduodenectomy.

### 2.4. Patient Data Analysis

Clinical patient data were collected and PFS was determined as the time between resection and radiologically confirmed cancer progression. Progressive disease was defined as cancer recurrence after resection, that could be either in lymph nodes, the peritoneal cavity, liver, lungs, and bones, with or without local recurrence in the pancreas. Statistical analyses were performed in SPSS Statistics 25 (IBM). The Ct values were processed in Excel and converted to fold expression after surgery using 2ˆ-ΔCt, normalized to the average expression of reference miR-29c-5p and miR-421. The pre- and post-surgery serum miR expression values were presented and analyzed in Prism Graphpad 5.01 and compared between three patient groups one-way ANOVA: patients with short PFS (0–8 months), median PFS (8–16 months), and long PFS (>16 months). Furthermore, hazard ratios (HR) were calculated using a Cox regression model in SPSS, to assess the strength of the association between the expression levels of the validated miRs and PFS.

### 2.5. In Situ Hybridization (ISH) of Matched Patient FFPE Pancreatic Cancer Tissues

The matched pancreaticoduodenectomy tissue specimens were collected at the Erasmus Medical Center for diagnostic evaluation. Residual FFPE material was used for biomarker analysis. Four-μm thick tissue sections on extra adhesive glass slides (Leica, Biosystems) were processed in the Discovery Ultra instrument (Ventana, Roche). The following automated Discovery Universal protocol was used: tissues were preheated at 70 °C for 4 min then deparaffinized at 70 °C for 12 min. Pretreatment was performed with CC1 for 16 min (cat. no. 950–224, Ventana). One drop of DISC inhibitor (cat. no. 760–4840, Ventana) was applied and incubated for 12 min. The 3’ and 5’-DIG labeled miRCURY LNA miRNA Detection probes (hsa-miR-125b-5p cat. no. YD00611756-BCG; hsa-miR-99a-5p cat. no. YD00619276-BCG; positive control hsa-U6 cat. no. YD00699002-BCG and negative control Scramble-miR cat. no. YD00699004-BCG, from Qiagen) were diluted in formamide-free MiRCURY LNA miRNA ISH Buffer (Qiagen cat. no. 339450) to a final 20 nM concentration, applied to the slides and incubated for 8 min. Denaturation was established at 90 °C for 8 min, followed by hybridization for 1 h (at 55 °C for miR-125b-5p; 53 °C for miR-34a-5p; 52 °C for miR-99a-5p; 54 °C for U6 and at 57 °C for the Scramble-miR). Slides were washed twice with SCC (DISCOVERY Ribowash 1× cat. no. 760–105, Ventana) and heated to 55 °C for 8 min. Slides were washed and heated again to 55 °C for 8 min. One drop of anti-DIG HRP enzyme conjugate (cat. no. 760–4822, Ventana) was applied and incubated for 16 min. Discovery amplification was performed using one drop of DISC AMP TSA BF and one drop of DISC AMP H2O2 BF (cat. no. 760–226, Ventana) for 32 min of incubation. One drop of DISC anti-BF HRP (cat no.760–4828, Ventana) was incubated 16 min, followed by one drop of DISC Ag C silver (cat. no. 760–227, Ventana) incubation for 16 min. The tissues were counterstained with Hematoxylin II (cat. no. 790–2208, Ventana) for 8 min, followed by incubation with Bluing Reagent Post Counterstain (cat. no. 760–2037, Ventana) incubation for 4 min. Adjacent tissue sections were stained with hematoxylin and eosin (H&E). The slides were scanned using the Nanozoomer 2.0-HT slide imager (Hamamatsu).

### 2.6. Immunohistochemistry in Human FFPE Pancreatic Cancer Tissues

Immunohistochemistry was performed with an automated immunohistochemistry staining system (Ventana BenchMark ULTRA, Ventana Medical Systems) using the 3,3′-diaminobenzidine method. In brief, following deparaffinization and heat-induced antigen retrieval for 64 min, the tissue sections were incubated for 32 min at 36 °C with either a mouse monoclonal antibody against the macrophage marker CD68 (clone KP-1 from cat. no. 790–2931, Ventana), or a rabbit monoclonal antibody raised against human immunoglobulin-associated alpha (CD79A), also known as B-Cell Antigen Receptor Complex-Associated Protein Alpha Chain, clone EP82 from Cell Marque. A subsequent amplification step was followed by incubation with hematoxylin II counter stain for 8 min and then a blue-coloring reagent for 8 min according to the manufacturer’s instructions (Ventana). The slides were scanned using the Nanozoomer 2.0-HT slide imager (Hamamatsu).

## 3. Results

### 3.1. Screening for Changes in Serum miRs Associated with PDAC Progression in Mice and Patients

**Figure 1.**
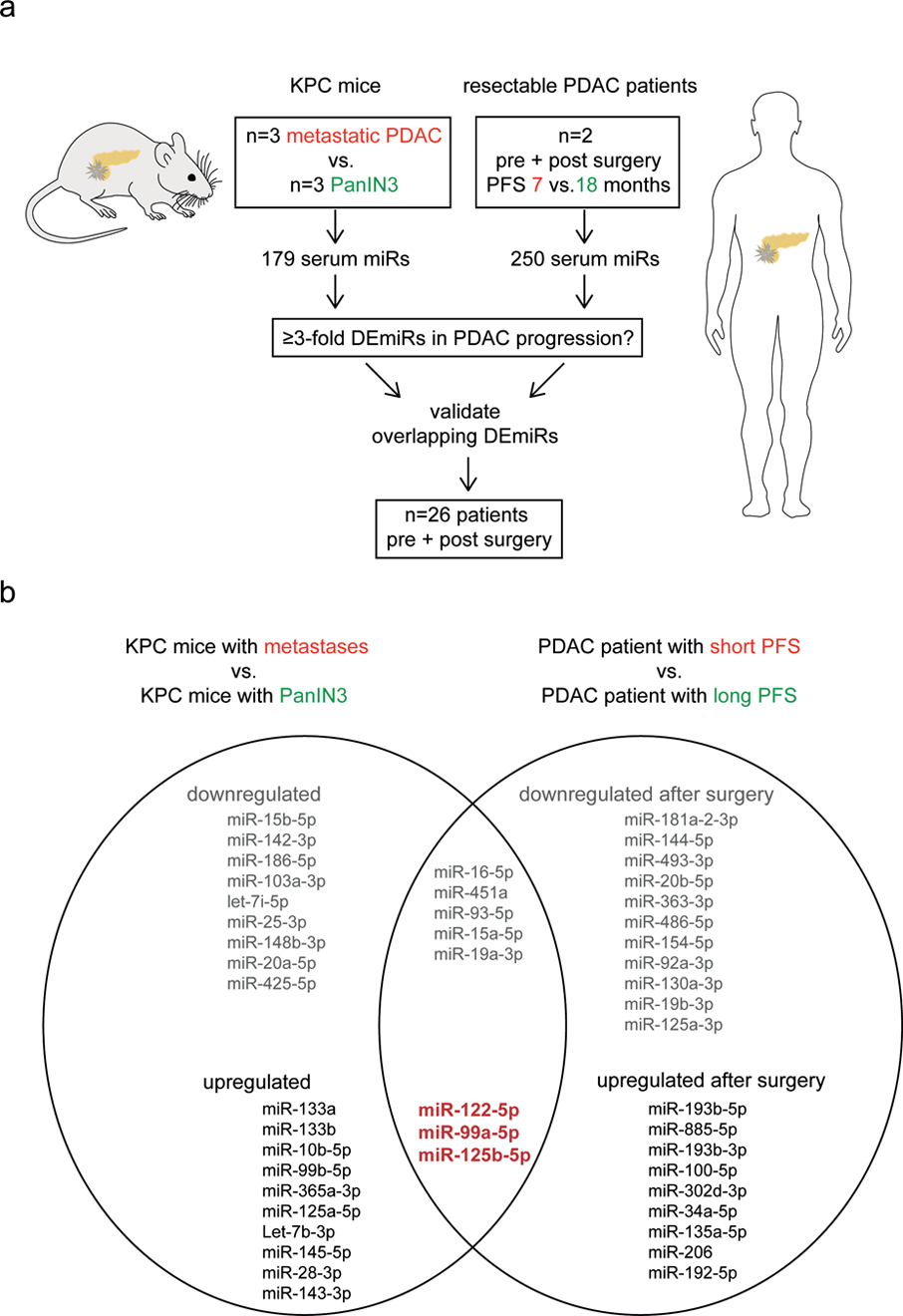
Overview of the serum miR screening in mice and patients to identify miRs associated with pancreatic cancer progression. (**a**) Schematic of performed RT-qPCR based miR discovery. Serum miRs from two groups of *LSL-Kras*^G12D/+^; *LSL-Trp53*^R172H/+^; *P48-Cre* (KPC) mice with different disease stages were analyzed for association with PDAC progression. Additionally, two patients with resectable PDAC were screened for expression of serum miRs to identify indicators of progression free survival (PFS) after surgical tumor removal. Overlapping differentially expressed miRs (DEmiRs) were selected for validation in an additional cohort of patients. (**b**) List of ≥3-fold DEmiRs that are associated with PDAC progression in KPC mice (left circle) and patients (right circle), identified in the miR screening. Upregulation of the miRs in bold red were associated with progressive PDAC in both mice and patients. These miRs were selected for validation.

The aim of our study was to determine which circulating miRs are associated with PDAC progression in mice as well as in patients. The approach of the miR screening is shown in Figure 1a.

The *LSL-Kras*^G12D/+^; *LSL-Trp53*^R172H/+^; *P48-Cre* (KPC) transgenic mice develop metastatic PDAC that recapitulates the human disease [23]. We first investigated the serum miR expression in mice with pancreatic pre-invasive intraepithelial neoplasia (PanIN) lesions (Figure S1a) and mice with metastatic PDAC (Figure S1b) to assess whether serum miRs are altered during cancer progression. Expression levels of 154 out of 179 serum miRs were detected above threshold and 27 miRs were differentially expressed ≥3-fold between the two mouse groups. KPC mice with metastatic disease had 13 miRs upregulated and 14 serum miRs downregulated compared to age-matched mice with only preinvasive lesions (Table S1).

Next, we analyzed paired pre- and post-pancreaticoduodenectomy sera from two patients with resectable PDAC. Our aim was to assess whether the change in serum miR expression after surgery was distinct in patients with different progression free survival (PFS) and compare the results to the murine metastases-related miRs. We initially profiled 250 miRs before and after primary tumor resection in two patients with PFS of 7 or 18 months (Figure 1a). From the 250 miRs we could reliably detect 171 serum miRs in all four samples, and we compared the changes in miR expression due to tumor resection of the patient with early recurrence to the changes in the patient with late recurrence. As a result of tumor resection, 28 miRs were differentially expression between the two patients: 12 miRs were upregulated ≥3-fold in the patient with fast progressive disease, whereas 16 miRs were downregulated compared to the patient with late progressive disease (Table S2). Figure 1b provides an overview of the PDAC progression-related serum miRs that were identified in our screening. Eight miRs (miR-16–5p, miR-451a, miR-93–5p, miR-15a-5p, miR-19a-3p, miR-122–5p, miR-99a-5p, and miR-125b-5p) were altered ≥3-fold in PDAC progression in mice as well as in patients. The three upregulated miRs that are associated with PDAC progression across species (miR-125b-5p, miR-99a-5p, and miR-122–5p, Figure 1b, in red), were reduced >4 fold after resection in the patient with long PFS, whereas these miRs were upregulated after surgery in the patient with early disease recurrence. We selected these three potentially oncogenic miRs for validation in 26 additional PDAC patients.

### 3.2. Circulating miR-125b-5p and miR-99a-5p Levels after Tumor Resection are Associated with Early Recurrence in PDAC Patients

**Figure 2.**
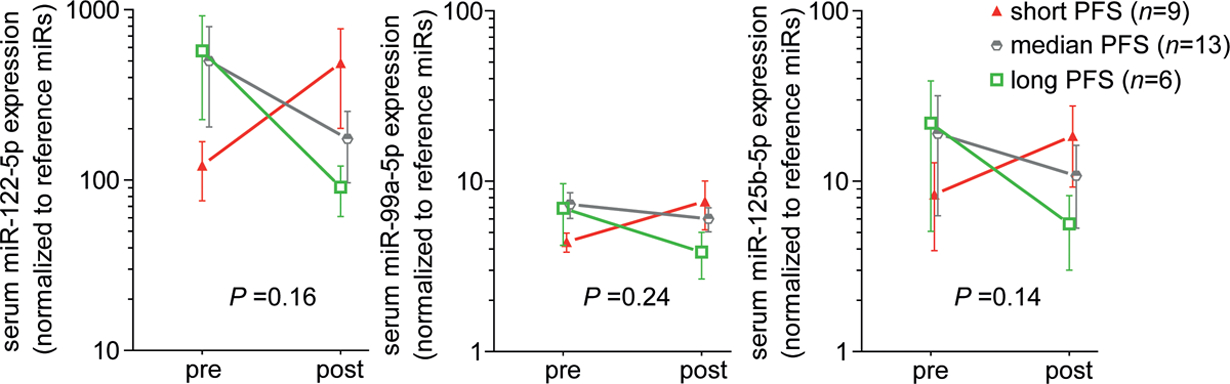
Pre- and post-resection expression levels of three serum miRs in PDAC patients with different PFS. Average expression levels of serum miRs 122–5p; 99a-5p or 125b-5p relative to the expression of two reference miRs, before and after surgery in three groups of patients. Short PFS in red = 0–8 months; median PFS in gray = 8–16 months; long PFS in green >16 months. Note the log scale of the *y*-axis. Error bars are SEM, *P*-values by one-way ANOVA comparing the three patient groups.

We measured the expression levels of miR-122–5p, miR-99a-5p, and miR-125b-5p before and after pancreaticoduodenectomy in the additional PDAC patients. Table 1 provides an overview of the patient characteristics of the total cohort. The PFS of the patients did not correlate with the resection margin: patients with R0 resection had a median PFS of 11.8 months (3.1–20.6 months 95% CI), whereas patients with R1 resection had a median PFS of 10.3 months (8.2–12.5 months 95% CI). We compared the miR levels upon tumor resection in the total patient cohort (N = 28), divided in three groups: *n* = 9 with short PFS (<8 months); *n* = 13 with median PFS (8–16 months); and *n* = 6 with long PFS (>16 months). The average expression levels of miR-122–5p, miR-99a-5p, and miR-125b-5p before and after surgery are shown in Figure 2. Although the differential changes in expression of miR-122–5p, miR-99a-5p, and miR-125b-5p between the three groups did not reach statistical significance, the serum expression of these three miRs show a trend of upregulation after tumor resection in patients with short PFS, and a trend of downregulation after tumor resection in patients who have a long PFS of >16 months. On the other hand, when analyzing the association of the serum miR levels with PFS by using a Cox regression model, two serum miRs after resection (miR-99a-5p and miR-125b-5p) were identified as significantly associated with shorter PFS (HR 1.73 and HR 1.34 respectively), shown in Table 2. In summary, we found that miR-125b-5p and miR-99a-5p are circulating prognostic indicators in KPC mice and patients with pancreatic cancer.

### 3.3. MiR-125b-5p and miR-99a-5p are Highly Expressed in Cells within Human Pancreatic Cancer Stroma

**Figure 3.**
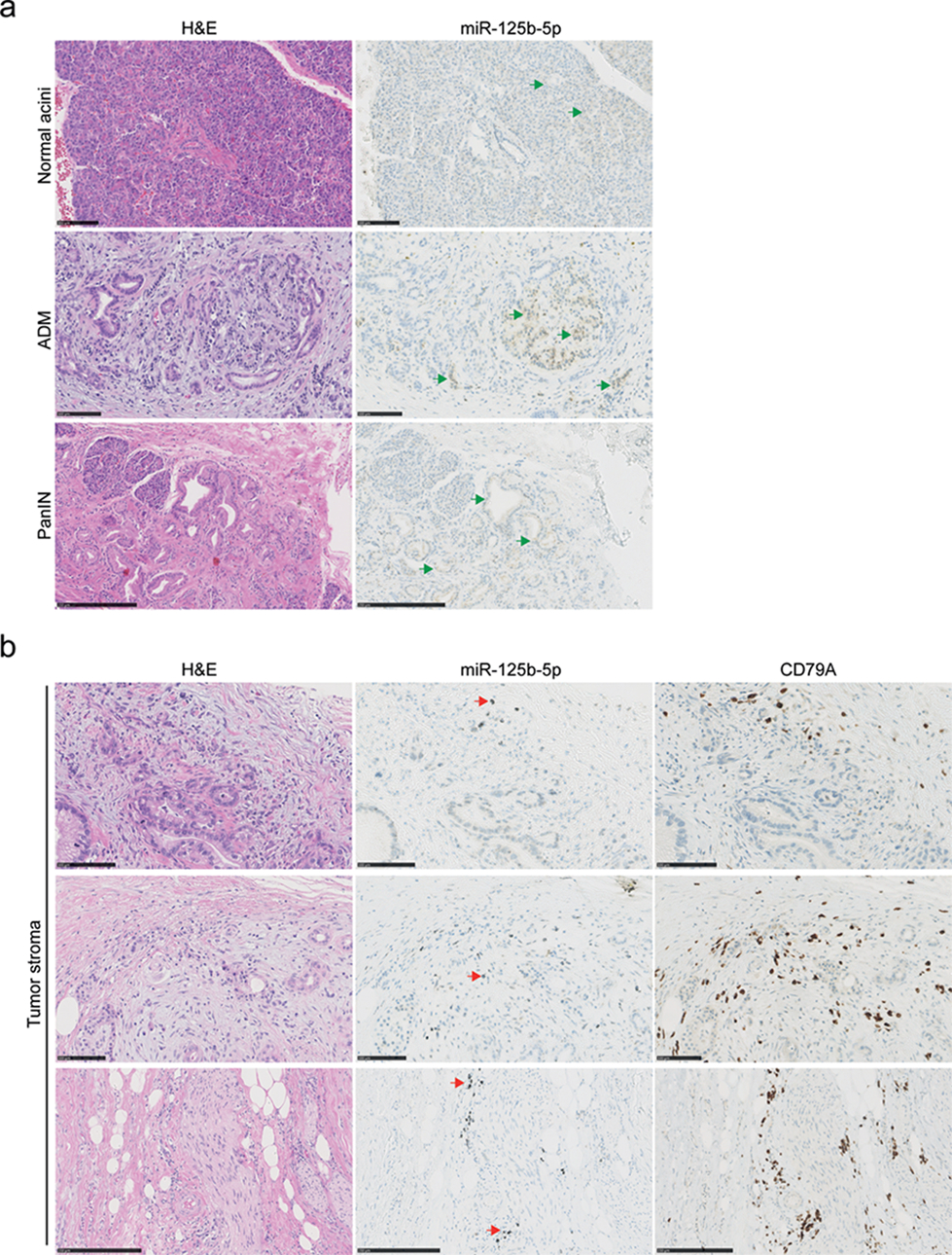
MiR-125b-5p detection by in situ hybridization in pancreatic cancer tissues. Serial FFPE tissue sections were stained with H&E or a DIG labeled miR-125b-5p probe. MiR-125b-5p expression is detected by silver staining resulting in brown/black coloring, whereas nuclei are stained with hematoxylin in blue. (**a**) Representative images of low expression of miR-125b-5p, (green arrows), in untransformed pancreatic acinar cells and cells that underwent acinar to ductal metaplasia (ADM) or pancreatic intraepithelial neoplasia (PanIN). Scale bars are 100 μm in the normal acini and ADM; scale bar is 250 μm in the PanIN. (**b**) Representative images of cells in the tumor stroma with high expression of miR-125b-5p (red arrows). Corresponding tissue sections are stained with H&E or the B-lymphocyte lineage marker CD79A antibody by immunohistochemistry (brown). Scale bars are 100 μm in the top two rows; scale bars are 250 μm in the bottom row.

In order to assess the expression of miR-125b-5p and miR-99a-5p in the tumors, we analyzed the paired resected pancreatic cancer tissues by in situ hybridization (ISH) with DIG-labeled locked nucleic acid miR probes. The positive and negative controls for the ISH assays are shown in Figure S2. MiR-125b-5p is expressed at low levels in the cytoplasm of normal pancreatic acinar cells, as well as in a fraction of the PanIN cells (Figure 3a). However, a small number of cells in the fibrotic stroma surrounding the invasive cancer cells express high levels of miR-125b-5p (Figure 3b). The stainings did not show a difference in the miR-125b-5p-high stromal cells in tumors from patients with short versus long PFS. Interestingly, the miR-125b-5p-high cells are localized in inflammatory cell aggregates in the tumor stroma. The miR-125b-5p-high cells are in low abundance, and resemble large immune cells such as macrophages or plasma cells. Immunohistochemistry shows that the miR expressing cells do not overlap with CD68 expressing macrophages (Figure S3). Instead, the miR expressing cells are located within clusters of CD79A positive cells (Figure S3, Figure 3b), indicating that miR-125b-5p may play a role in B-lymphocyte/plasma cell infiltration in pancreatic cancer stroma.

**Figure 4.**
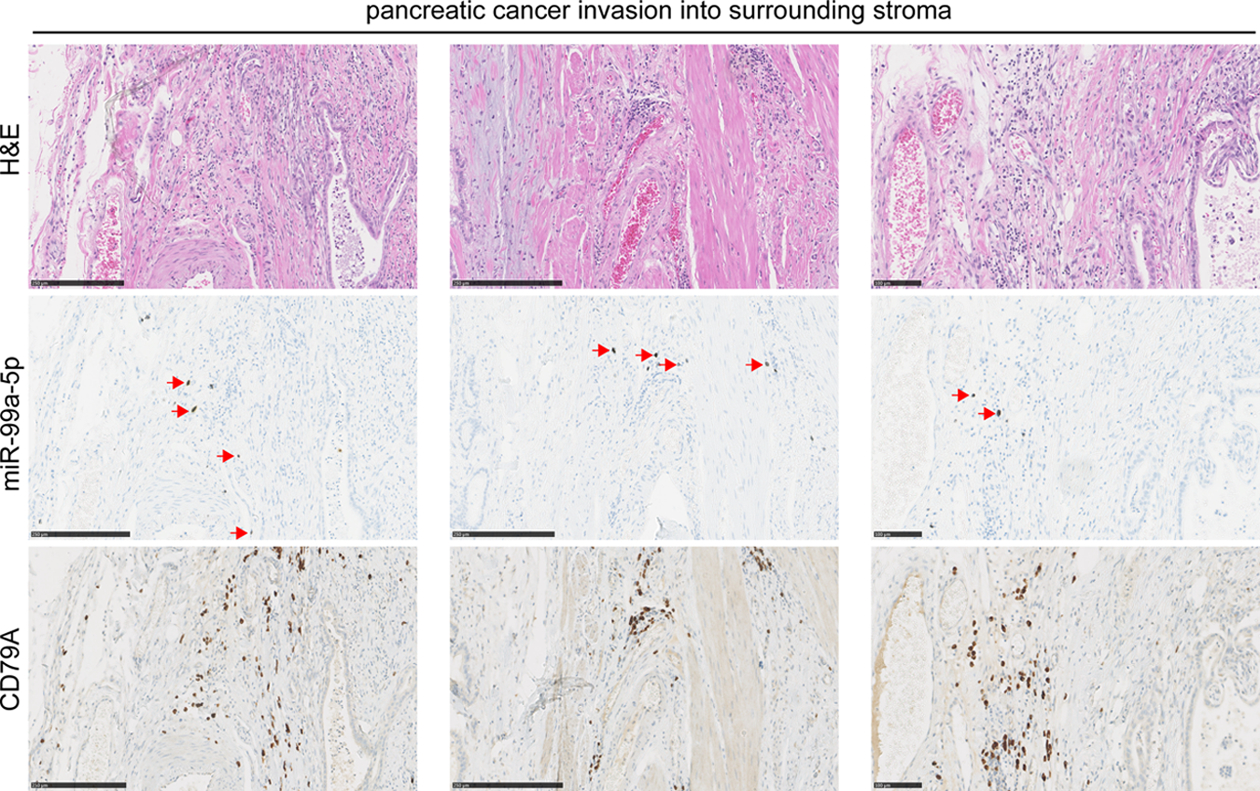
MiR-99a-5p detection by in situ hybridization in pancreatic cancer tissues. Serial FFPE tissue sections were stained with H&E a DIG labeled miR-99a-5p probe, or the B-lymphocyte lineage marker CD79A antibody by immunohistochemistry (brown). Red arrows indicate miR-99a-5p expression, detected with silver staining resulting in brown/black coloring, whereas cell nuclei are stained with hematoxylin in blue. Scale bars are 250 μm in the first two columns; scale bar is 100 μm in the right-most column.

MiR-99a-5p expressing cells are less abundant than miR-125b-5p positive cells in the pancreatic tumors. MiR-99a-5p positive cells are only present in limited regions of tumor stroma in a few of the tumors, and are located in muscular and connective tissue where pancreatic cancer cells invade the stroma. Figure 4 shows tumor regions with cells that express high levels of miR-99a-5p. In the centers of the pancreatic tumors rich in epithelial cancer cells, there are no cells that express miR-99a-5p. When comparing the location of CD79A positive cells in the adjacent tissue slides, we observed that the miR-99a-5p expressing inflammatory cells are also in close proximity to cells of the B-cell lineage (Figure 4). The abundance of inflammatory cells that express CD79A protein, or high levels of miR-125b-5p or miR-99a-5p in the resected tumor tissues was not different in patients with short versus long PFS, although a single section may not represent the overall extent of expression across an entire tumor.

In summary, we found that serum miR-125b-5p and miR-99a-5p expression levels are correlated with PDAC progression in transgenic mice as well as in patients after surgical tumor removal. MiR-125b-5p and miR-99a-5p are highly expressed in a subset of inflammatory cells in the pancreatic tumor stroma, associated with cells from the B-lymphocyte lineage.

## 4. Discussion

Approved circulating biomarkers for the prognostication of patients with pancreatic cancer are currently lacking. Clinical follow-up of pancreatic cancer patients in the Netherlands is not standardized [27] and currently consists of physical examination and at times radiographical imaging by CT scans which have a low performance in detecting early metastatic disease. Carbohydrate antigen 19–9 (CA19–9) is a sialylated Lewis blood group antigen associated with different cancers, including PDAC [28] and could serve as biomarker for progressive disease [29]. However, the sensitivity is low and up to 20% of the population is Lewis negative and cannot synthesize CA19–9 [30].

We sought to identify circulating biomarkers that can indicate high risk of fast recurrence shortly after resection, before metastases are visible on CT scan. Repeated blood draws followed by miR expression analysis that could stratify patients at higher risk for progressive disease and could prompt treatment adjustment would improve patient care. MiRs are suitable candidates for the prediction of cancer progression due to their altered expression during tumorigenesis and their stability in the circulation [22]. Numerous studies have shown that circulating miRs are altered in early PDAC as well as in metastatic PDAC [31]. Zhou et al. showed that patients with early PDAC stage could be distinguished from healthy controls by the upregulation of circulating miR-122–5p; miR-125b-5p; miR-192–5p; miR-193b-3p; miR-122–3p; and miR-27b-3p [14]. In contrast, they found that among the PDAC patients, decreased miR-125b-5p levels before treatment are associated with worse overall survival. Also, increased serum expression of the miR-99 family is recently found associated with the diagnosis of pancreatic cancer [32]. Studies similar to these, which assess circulating miR levels for the diagnosis of PDAC, are difficult to corroborate. Inconsistent results are due to interpatient diversity as well as technical differences in quantitation and data normalization [33].

Unfortunately, a subset of patients with PDAC that undergo primary tumor resection rapidly succumb to disease recurrence, whereas other patients have longer progression free survival after surgery. Identifying the patients at risk for early disease recurrence could prompt adjustments in adjuvant treatment decisions and thus improve patient outcome. In the current study, we performed a prognostic miR biomarker analysis using serum samples from treatment-naive patients with surgically resectable PDAC. We profiled serum miR expression before and after surgery and compared the changes to the PFS of the patients. Our comparison shows the changes in serum miR levels after removal of the primary cancer. The aim was to select biomarkers that could identify patients at risk, shortly after resection, before metastases are visible on CT scan. Therefore, no serum from the patients included here was collected at time of radiographically visible metastatic disease.

Genetically engineered mouse models of pancreatic cancer have led to major improvements in the understanding of PDAC development. Specifically, the *LSL-Kras*^G12D/+^; *LSL-Trp53*^R172H/+^; *P48-Cre* mouse model indicated by the acronym ‘KPC’ succumbs to progressive PDAC that mimics the features of the human disease [23]. KPC mice initially develop preinvasive acinar to ductal metaplasia (ADM) and pancreatic intraepithelial neoplasia (PanIN) lesions before widespread PDAC, all in the presence of an intact immune system. The median survival of KPC mice is 5 months and all mice succumb to the disease before the age of one year [23]. We performed a cross-species comparison of serum miR expression: the prognostic miRs we identified in the patients shortly after surgery were also correlated to miRs in KPC mice with PDAC metastases, confirming their significance in pancreatic cancer progression. Most murine miRs are homologues to human miRs and share the same sequence. We made this comparison across species given the notion that patients who develop radiographically visible cancer lesions within 8 months after tumor resection are considered to have occult metastatic disease at the time of surgery.

Circulating miRs that are associated with cancer presence often do not originate from cancer cells themselves. For example, miR-125b expression in colorectal liver and lung metastases is ~3 fold and ~7-fold higher in the stroma than in the cancer cells [34]. Indeed, the majority of circulating miRs are derived from blood cells and the endothelium [35,36]. PDAC progression goes hand in hand with alterations in systemic immune cell profiles [37] and this link between altered circulating miRs during cancer progression and immune cells was confirmed in our study. We found that miR-125b-5p and miR-99a-5p levels are high in cells that are closely associated with B-lymphocytes in the tumor stroma. Others have shown that miR-125b-5p is upregulated in B-lymphocytes [38] and can cause leukemia in mice [39,40]. In pancreatic tumors, high levels of infiltrating plasma cells are significantly correlated with worse prognosis in terms of overall survival in patients after surgery [41].

We found that after surgical tumor removal, patients with high levels of serum miR-125b-5p or miR-99a-5p have shorter PFS. MiR-125b-5p and miR-99a-5p belong to the 20 most abundant miRs in human plasma exosomes [42], indicating that these miRs are actively packaged and released into the bloodstream. Circulating miRs are transferred from cell to cell and can elicit immune modulation [43]. Serum miRs interact more with immune-related mRNA genes that with non-immune related genes [44]. MiR-containing T-regulatory-cell-derived exosomes suppress pathogenic T helper 1 cells [45]. A recent study showed that miR-125b-5p and miR-99a-5p both downregulate the activation of γδ T-lymphocytes and their cytotoxicity towards lymphoma cells [46]. In humans and rats, treatment with methylprednisolone leads to increased plasma miR-99a-5p levels, suggesting miR-99a-5p is involved in systemic immune suppression [47]. Others have shown that miR-99a-5p inhibits the mammalian target of rapamycin (mTOR) signaling in bladder cancer cells [48], which was also described in gastric cancer tissues by Zhang et al. [49]. mTOR is not only an important cancer-related pathway, but also crucial for hematopoietic cell fate [50,51], since the differentiation of naive T-cells into distinct effector T-cells is promoted by mTOR [50]. In the absence of mTORC1 activity myeloid differentiation is impaired due to a block in glucose uptake and lipid metabolism [52]. Cell-free miR-99a-5p levels are high in the blood of patients with progressive PDAC, suggesting that the miR is produced at high levels by a subset of leukocytes, and can potentially be taken up by other cells, leading to the abrogation of mTOR among other pathways.

On the other hand, expression of miR-122–5p is liver specific and absent in most other tissues [53–55]. In the KPC mice with metastatic PDAC, miR-122–5p was upregulated 22-fold in comparison to mice with pre-invasive lesions. In the patients who underwent surgical tumor resection, serum miR-122–5p levels went up after surgery in patients with early disease recurrence, whereas post-surgery serum miR-122–5p levels did not distinguish patients based on PFS, or based on the presence of liver metastases. This may suggest that liver damage is present at some level in all patients who undergo pancreaticoduodenectomy for PDAC.

There has been very little to no research that compares the levels of miRs after surgical removal of primary pancreatic tumors. In our study, we compared serum miRs pre- and post-resection in treatment-naive patients that were treated at the Erasmus Medical Center in the Netherlands between 2013 and 2017. Recent studies show that patients with (borderline) resectable PDAC that undergo preoperative chemo/radiotherapy may have improved survival [56–59]. From now on, all patients with (borderline) resectable PDAC in the Netherlands will be offered to receive preoperative chemotherapy (with or without radiotherapy) in the context of a clinical trial (PREOPANC-2 trial), if the performance status of the patient permits and the patient is willing to undergo systemic treatment. Chemotherapy has a vast impact on the immune landscape [60,61]. Whether serum miR-125b-5p and miR-99a-5p are still predictive of disease progression after surgery in pre-treated patients remains to be evaluated. Therefore, we are currently collecting blood samples to evaluate the changes in the serum miRs of patients with (borderline) resectable tumors who undergo pre-operative FOLFIRINOX chemotherapy or gemcitabine-based chemoradiation.

In summary, we found that serum miR-125b-5p and miR-99a-5p levels in patients with PDAC are potential indicators of early disease recurrence after surgery and are likely originating from immune cells. This is a pilot study, the reliability and validity of these circulating biomarkers to accurately detect early PDAC recurrence needs to be validated in a larger independent cohort. Moreover, performing dual stainings for miR expression by ISH and immune related protein expression by immunohistochemistry is one of our future directions. Further dissection of the dynamics and functions of miR-125b-5p and miR-99a-5p in the immune response to pancreatic cancer will provide fundamental information to assist in the development of biomarkers and better immune therapies.

## Supplementary Material

Suppl Fig S1 **Figure S1.** Different stages of pancreatic neoplasia in KPC mice at the age of 5 months. (a) Images of formalin fixed, paraffin embedded (FFPE), H&E stained pancreatic tissues obtained from *LSL-Kras*^G12D/+^; *LSL-Trp53*^R172H/+^; *P48-Cre* (KPC) mice at the age of 5 months. PanIN-3 lesions are indicted by the green arrows. 20X magnification, scale bar = 100 μm. (b) Images of FFPE, H&E stained liver and lung tissues from KPC mice at the age of 5 months. Metastatic pancreatic cancer lesions are indicated by the black arrows. 10X magnification, scale bar = 100 μm

Suppl Fig S2 **Figure S2.** MiR in situ hybridization (ISH) controls. Images of FFPE tissues that are stained with silver after in situ hybridization of dual-DIG labeled miR probes leading to brown/black staining. Nuclei are stained with Hematoxylin in blue. (a) U6 expression in human colon and testis tissue, serve as positive controls for the ISH assay. Scale bars = 250 μm. (b) MiR-125b-5p expression in Her2Neu positive human breast cancer and human testis tissues. Note the distinct staining patterns in the breast cancer cells and Sertoli cells that show low miR-125b-5p expression (green arrows) versus the single cells in the stroma that have high miR-125b-5p expression (red arrows). Scale bars = 250 μm. (c) MiR-99a-5p expression in human pancreatic cancer and liver. Cells in the stroma with high levels of miR-99a-5p are indicated by the red arrows. (d) Scramble miR expression in human colon and testis tissues, serves as a negative control for the ISH assay

Suppl Fig S3 **Figure S3.** MiR-125b-5p, CD79A and CD68 expression in human PDAC tumor stroma. Serial FFPE tissue sections were stained with H&E, a DIG labeled miR-125b-5p probe (black staining indicated by red arrows), a B-lymphocyte lineage marker CD79A antibody, or the macrophage marker CD68 antibody by immunohistochemistry (brown). Scale bars = 100 μm

Suppl. Table 1 **Table S1.** Murine serum miRs associated with PDAC progression. Differentially expressed miRs (DEmiRs) ≥3-fold in KPC mice with pancreatic cancer metastases versus mice with pre-invasive PanIN-3 lesions

Suppl. Table 2 **Table S2.** Human serum miRs associated with PDAC progression after surgery. Differentially altered miRs (≥3-fold) upon surgery in a patient with early disease progression compared to a patient with late disease progression.

## Figures and Tables

**Table 1.** Patient characteristics. Patients who underwent pancreaticoduodenectomy for pancreatic cancer and were included for serum miR analysis.

	all patients (n=28)
Age (years) median (range)	71.9 (35.5)
Sex (%) Male Female	22 (78.6) 6 (21.4)
T-stage * (%) 1 2 3 4	0 (0.0) 0 (0.0) 28 (100.0) 0 (0.0)
N-stage * (%) 0 1	5 (17.9) 23 (82.1)
Resection margin ** (%) R0 R1 unknown	13 (46.4) 14 (50.0) 1 (3.6)
Adjuvant Chemotherapy (%)	22 (78.6)
PFS (months) median (IQR)	10.7 (6.7–15.8)
OS (months) median (IQR)	16.0 (10.2–31.6)

* TNM classification Pancreas (8th edition UICC)

** R0 >1 mm; PFS = progression free survival; IQR = inter quartile range; OS = overall survival.

**Table 2.** Hazard ratios (HR) for PDAC recurrence after resection according to serum miR expression levels.

Serum miR expression		HR (95% CI)		*P*-value	
Pre surgery in miR-122–5p		0.941 (0.805–1.100)		0.444	
Change in miR-122–5p		1.151 (0.982–1.349)		0.082	
Post surgery miR-122–5p		1.107 (0.901–1.361)		0.334	
		
Pre surgery miR-125b-5p		1.016 (0.821–1.256)		0.887	
Change in miR-125b-5p		1.246 (0.984–1.577)		0.068	
Post surgery miR-125b-5p		**1.341** (1.043–1.723)		**0.022**	
		
Pre surgery miR-99a-5p		0.999 (0.694–1.440)		0.997	
Change in miR-99a-5p		**1.402** (1.003–1.961)		**0.048**	
Post surgery miR-99a-5p		**1.734** (1.098–2.740)		**0.018**	

miR = microRNA; CI = confidence interval.
