# Supplementary material for: Immune-Related Circulating miR-125b-5p and miR-99a-5p Reveal a High Recurrence Risk Group of Pancreatic Cancer Patients after Tumor Resection
Source: Appl Sci (Basel). Author manuscript; Available in PMC 2021 Sep 3. (PMC8415800; doi:10.3390/app9224784)
Supplement: Suppl. Table 1 — Table S1. Murine serum miRs associated with PDAC progression. Differentially expressed miRs (DEmiRs) ≥3-fold in KPC mice with pancreatic cancer metastases versus mice with pre-invasive PanIN-3 lesions [file NIHMS1733585-supplement-Suppl__Table_1.docx]

| **Upregulated miRs** | | **Downregulated miRs** | |
| --- | --- | --- | --- |
| Serum miR | Fold difference | Serum miR | Fold difference |
| miR-122-5p | 22.63 | miR-15a-5p | 0.13 |
| miR-125b-5p | 8.22 | miR-451a | 0.13 |
| miR-133a | 5.94 | miR-15b-5p | 0.16 |
| miR-133b | 5.82 | miR-142-3p | 0.17 |
| miR-10b-5p | 5.28 | miR-186-5p | 0.24 |
| miR-99b-5p | 5.21 | miR-103a-3p | 0.26 |
| miR-365a-3p | 5.17 | let-7i-5p | 0.27 |
| miR-125a-5p | 5.13 | miR-25-3p | 0.29 |
| miR-99a-5p | 5.03 | miR-93-5p | 0.30 |
| let-7b-3p | 4.26 | miR-16-5p | 0.31 |
| miR-145-5p | 3.86 | miR-19a-3p | 0.31 |
| miR-28-3p | 3.46 | miR-148b-3p | 0.32 |
| miR-143-3p | 3.41 | miR-20a-5p | 0.33 |
|  | | miR-425-5p | 0.33 |

**Supplementary Table S1. Murine serum miRs associated with PDAC progression.** Differentially expressed miRs (DEmiRs) in KPC mice with pancreatic cancer metastases versus mice with pre-invasive PanIN-3 lesions.
