# Supplementary material for: Immune-Related Circulating miR-125b-5p and miR-99a-5p Reveal a High Recurrence Risk Group of Pancreatic Cancer Patients after Tumor Resection
Source: Appl Sci (Basel). Author manuscript; Available in PMC 2021 Sep 3. (PMC8415800; doi:10.3390/app9224784)
Supplement: Suppl. Table 2 — Table S2. Human serum miRs associated with PDAC progression after surgery. Differentially altered miRs (≥3-fold) upon surgery in a patient with early disease progression compared to a patient with late disease progression. [file NIHMS1733585-supplement-Suppl__Table_2.docx]

| **Upregulated miRs** | | **Downregulated miRs** | |
| --- | --- | --- | --- |
| Serum miR | Fold difference | Serum miR | Fold difference |
| miR-193b-5p | 55.36 | miR-181a-2-3p | 0.05 |
| miR-885-5p | 27.22 | miR-144-5p | 0.16 |
| miR-122-5p | 23.95 | miR-493-3p | 0.16 |
| miR-193b-3p | 18.73 | miR-16-5p | 0.17 |
| miR-100-5p | 5.61 | miR-20b-5p | 0.18 |
| miR-99a-5p | 5.10 | miR-363-3p | 0.18 |
| miR-125b-5p | 4.97 | miR-486-5p | 0.19 |
| miR-302d-3p | 4.78 | miR-451a | 0.20 |
| miR-34a-5p | 4.57 | miR-93-5p | 0.20 |
| miR-135a-5p | 4.39 | miR-154-5p | 0.20 |
| miR-206 | 4.39 | miR-15a-5p | 0.25 |
| miR-192-5p | 3.31 | miR-92a-3p | 0.27 |
|  | | miR-19a-3p | 0.30 |
|  |  | miR-130a-3p | 0.31 |
|  |  | miR-19b-3p | 0.32 |
|  |  | miR-125a-3p | 0.32 |

**Table 2.** **Human serum miRs associated with PDAC progression after surgery.** Differentially altered miRs upon surgery in a patient with early disease progression compared to a patient with late disease progression.
